# Insights from the Genome Sequence of *Mycobacterium lepraemurium*: Massive Gene Decay and Reductive Evolution

**DOI:** 10.1128/mBio.01283-17

**Published:** 2017-10-17

**Authors:** Andrej Benjak, Tanvi P. Honap, Charlotte Avanzi, Enrique Becerril-Villanueva, Iris Estrada-García, Oscar Rojas-Espinosa, Anne C. Stone, Stewart T. Cole

**Affiliations:** a Global Health Institute, École Polytechnique Fédérale de Lausanne, Lausanne, Switzerland; b School of Life Sciences, Arizona State University, Tempe, Arizona, USA; c Departamento de Psicoinmunología, Instituto Nacional de Psiquiatría Ramón de la Fuente, Mexico City, Mexico; d Departamento de Inmunología, Escuela Nacional de Ciencias Biológicas, Instituto Politécnico Nacional, Mexico City, Mexico; e School of Human Evolution and Social Change, Arizona State University, Tempe, Arizona, USA; f Center for Evolution and Medicine, Arizona State University, Tempe, Arizona, USA; Institut Pasteur; Washington University in St. Louis School of Medicine

**Keywords:** *Mycobacterium lepraemurium*, comparative genomics, genome sequencing, murine leprosy

## Abstract

*Mycobacterium lepraemurium* is the causative agent of murine leprosy, a chronic, granulomatous disease similar to human leprosy. Due to the similar clinical manifestations of human and murine leprosy and the difficulty of growing both bacilli axenically, *Mycobacterium leprae* and *M. lepraemurium* were once thought to be closely related, although it was later suggested that *M. lepraemurium* might be related to *Mycobacterium avium*. In this study, the complete genome of *M. lepraemurium* was sequenced using a combination of PacBio and Illumina sequencing. Phylogenomic analyses confirmed that *M. lepraemurium* is a distinct species within the *M. avium* complex (MAC). The *M. lepraemurium* genome is 4.05 Mb in length, which is considerably smaller than other MAC genomes, and it comprises 2,682 functional genes and 1,139 pseudogenes, which indicates that *M. lepraemurium* has undergone genome reduction. An error-prone repair homologue of the DNA polymerase III α-subunit was found to be nonfunctional in *M. lepraemurium*, which might contribute to pseudogene formation due to the accumulation of mutations in nonessential genes. *M. lepraemurium* has retained the functionality of several genes thought to influence virulence among members of the MAC.

## IMPORTANCE

*Mycobacterium lepraemurium* seems to be evolving toward a minimal set of genes required for an obligatory intracellular lifestyle within its host, a niche seldom adopted by most mycobacteria, as they are free-living. *M. lepraemurium* could be used as a model to elucidate functions of genes shared with other members of the MAC. Its reduced gene set can be exploited for studying the essentiality of genes in related pathogenic species, which might lead to discovery of common virulence factors or clarify host-pathogen interactions. *M. lepraemurium* can be cultivated *in vitro* only under specific conditions and even then with difficulty. Elucidating the metabolic (in)capabilities of *M. lepraemurium* will help develop suitable axenic media and facilitate genetic studies.

## OBSERVATION

Murine leprosy is a chronic, granulomatous disease caused by *Mycobacterium lepraemurium*. Murine leprosy mainly affects the skin, mucosa of the upper respiratory tract, and eyes; however, unlike human leprosy, the viscera are commonly affected, whereas the peripheral nerves are not (1). Murine leprosy was first reported in the early 20th century in rats in Ukraine (2), after which similar cases were reported from other countries (3, 4). *M. lepraemurium* is one of the causative agents of leprosy in cats and causes granulomatous skin lesions that often involve ulceration (5).

In humans, leprosy is primarily caused by *Mycobacterium leprae* and *Mycobacterium lepromatosis*. Numerous similarities exist between human and murine leprosy, including disease transmission through abrasions in the skin and the mucosal respiratory surfaces, a similar spectrum of disease progression, suppression of cell-mediated immunity, and strong humoral immunity (1). This led to the hypothesis that these species were closely related, and hence, it was thought that murine leprosy might serve as a model for human leprosy (6–8). DNA hybridization studies and analysis of the 16S rRNA gene sequences suggested that *M. lepraemurium* might actually be more related to the *Mycobacterium avium* complex (MAC) (9, 10). However, to date, no phylogenomic study of *M. lepraemurium* has been conducted, and the lack of a genome sequence has restricted our understanding of its biology and evolution.

**FIG 1:**
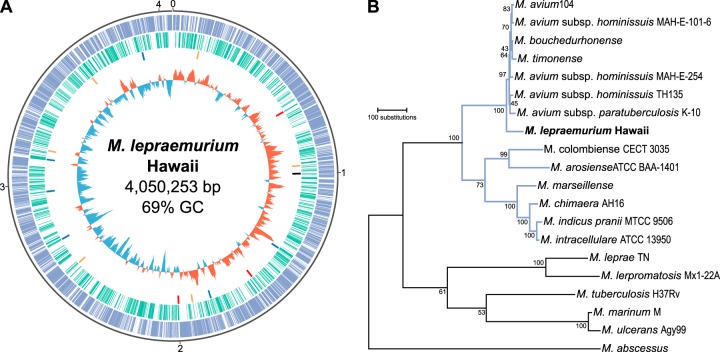
The genome of *Mycobacterium lepraemurium* strain Hawaii. (A) Graphical representation of the genome and its features. The origin of replication is at 12 o’clock, and the genome sequence runs clockwise. Ticks around the outermost circle mark million bases. Looking inwards, the outermost track or the first track (blue) shows functional genes. The second track (green) shows pseudogenes. The third track shows insertion sequences (all dysfunctional) colored to distinguish families (orange, red, black, and blue) and exaggerated in size for visibility. The fourth track shows the GC skew, calculated for a 20-kb window sliding every 1 kb, represented as a histogram with positive values pointing outward (red bars) and negative values pointing inward (blue bars). (B) Maximum parsimony tree of *M. lepraemurium* and selected mycobacterial species. The tree was created using MEGA7 from concatenated amino acid sequences (3,936 positions) of 11 proteins (DnaN, RplI, GrpE, MetG, RplY, PheT, FtsQ, HolA, MiaA, FtsY, and FtsX). Branches corresponding to the *Mycobacterium avium* complex are in blue. Bootstrap support, estimated from 500 replicates, is given below each branch. An expanded version of this tree, including additional genomes, is in Fig. S1 in the supplemental material.

Here, we describe the complete genome of *M. lepraemurium*, which was sequenced using single-molecule real-time (SMRT; Pacific Biosciences) and Illumina technologies. The *M. lepraemurium* genome was found to be circular, with a total GC content of 68.99%, and 4,050,523 bp in length. No plasmids were found. The genome comprises 3,821 “protein-coding” genes, of which 2,682 are functional genes and 1,139 are pseudogenes (Fig. 1A). *M. lepraemurium* belongs to the MAC and is more closely related to the *M. avium* clade than to the *M. intracellulare* clade (Fig. 1B and see Fig. S1 and S2 in the supplemental material).

10.1128/mBio.01283-17.2 FIG S1  Maximum parsimony tree of *M. lepraemurium* and other mycobacterial species. The tree was created using MEGA7 from concatenated amino acid sequences (3,948 positions) of 11 proteins (DnaN, RplI, GrpE, MetG, RplY, PheT, FtsQ, HolA, MiaA, FtsY, and FtsX). Bootstrap support, estimated from 500 replicates, is given below each branch. Download FIG S1, EPS file, 0.2 MB. Copyright © 2017 Benjak et al. 2017 Benjak et al. This content is distributed under the terms of the Creative Commons Attribution 4.0 International license.

10.1128/mBio.01283-17.3 FIG S2  Phylogeny of *M. lepraemurium* and selected mycobacterial species based on whole-genome sequence alignments. Species belonging to the *M. avium* complex are highlighted in blue and *M. lepraemurium* is denoted in red. *M. abscessus* was used as the outgroup. (A) Maximum likelihood tree. The tree was created using RAxML based on 460,625 variable nucleotide sites and a general time reversible (GTR) model with gamma distribution. Bootstrap support estimated from 100 replicates is given below each branch. (B) Neighbor-joining tree. The tree was created using MEGA7 based on 460,625 variable nucleotide sites and the p-distance method. Bootstrap support estimated from 500 replicates is given below each branch. (C) Maximum parsimony tree. The tree was created using MEGA7 based on 460,625 variable nucleotide sites and the SPR algorithm. Bootstrap support estimated from 500 replicates is given below each branch. Download FIG S2, EPS file, 0.2 MB. Copyright © 2017 Benjak et al. 2017 Benjak et al. This content is distributed under the terms of the Creative Commons Attribution 4.0 International license.

### Genome downsizing and pseudogene formation.

At 4.05 Mb, the *M. lepraemurium* genome is the smallest within the MAC and one of the smallest among mycobacteria (9th out of the 350 sequenced mycobacterial species). More strikingly, within the mycobacteria, only the *M. leprae* and *M. lepromatosis* genomes contain fewer functional protein-coding genes than the *M. lepraemurium* genome. The presence of 1,139 pseudogenes indicates that *M. lepraemurium* underwent reductive evolution, which is characteristic for strictly host-associated organisms. Analyses of pseudogene families within a diverse set of prokaryotes have shown that pseudogenes are most likely to occur in ABC transporter, short-chain dehydrogenase/reductase, sugar transporter, cytochrome P450, and proline-glutamate (PE)/proline-proline-glutamate (PPE) gene families (11). The *M. lepraemurium* genome was found to contain pseudogenes in all these families.

**FIG 2:**
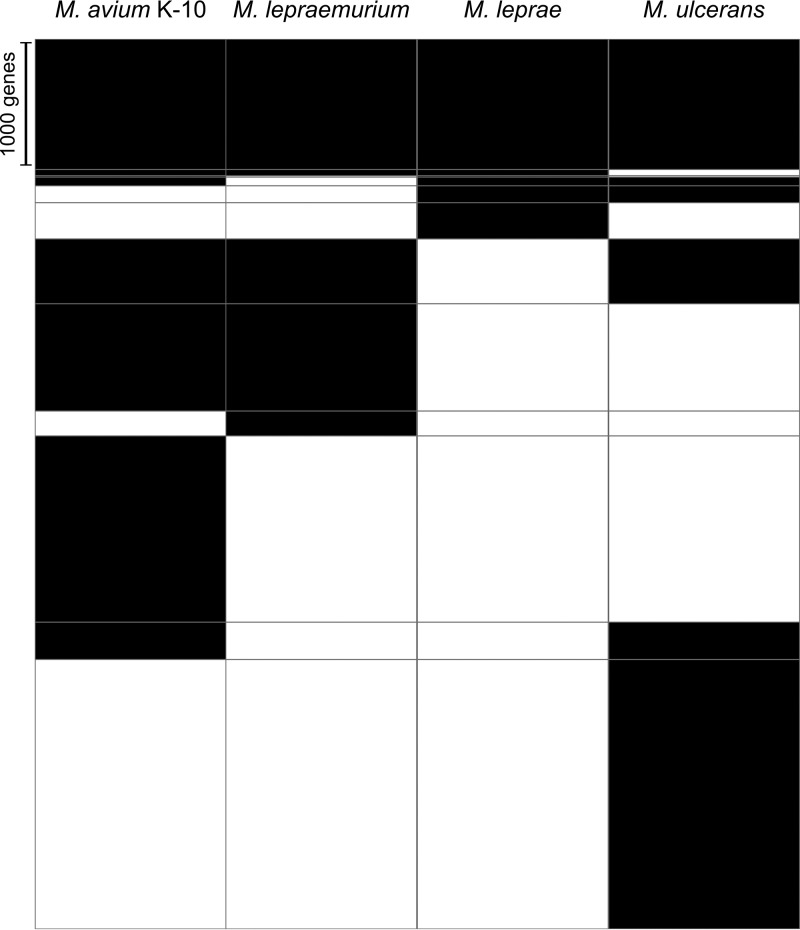
Heatmap of the gene orthology between *M. avium* subsp. *paratuberculosis* K-10, *M. lepraemurium*, *M. leprae*, and *M. ulcerans*. Genes are shown in black, and the absence of genes is shown in white. The raw height corresponds to the number of genes.

*M. lepraemurium* is the third mycobacterial species known to have undergone reductive evolution. The other species include the common ancestor of *M. leprae* and the closely related *M. lepromatosis*, which underwent genome reduction before the two species diverged, as well as *Mycobacterium ulcerans*, which is in a state of intermediate reductive genome evolution (12–14). Genome size among *M. avium* subspecies varies considerably (around 4.8 to 5.5 Mb), but no extensive pseudogenization was observed in these organisms. Curiously, *M. lepraemurium* seems to be evolving convergently toward a minimal gene set such as the one retained by *M. leprae* (Fig. 2). This may be due to both species having adapted to a similar niche, consequently resulting in similar pathological manifestations.

Loss of the DnaQ-mediated proofreading mechanism of DNA polymerase III α-subunit has been hypothesized as the cause of pseudogene formation in *M. leprae* (12), and a similar pattern has been observed in other obligate pathogens and symbionts (15, 16). In *M. lepraemurium*, a homologue of the error-prone repair DNA polymerase III α-subunit (*MLM_3495*) is nonfunctional, which might contribute to a higher error rate leading to pseudogene formation in this species.

The AT content of the pathogen/symbiont genome seems to correlate with the age of the strict association with the host (16). As expected, the genome of *M. leprae* has the highest AT content of all mycobacteria (42.2%), especially in pseudogenes (43.5%). On the other hand, the genome of *M. lepraemurium* is as AT-poor as that of its closest relative, *M. avium* (31%), and shows only a small difference in the AT content of functional genes and pseudogenes (30.46% and 31.05%, respectively). This suggests that *M. lepraemurium* adopted the “strictly intracellular” niche relatively recently, as evidenced by its phylogenetic relatedness to the free-living *M. avium* (Fig. S1). Genomic data for other *M. lepraemurium* strains would allow us to determine whether the pseudogene content varies between strains, which would indicate ongoing reductive evolution.

### Repetitive sequences and mobile elements.

Repeats and mobile elements are the major vehicles leading to genomic rearrangements and large deletions, and hence, they are thought to play an important role in genome downsizing. Yet, there seems to be a negative correlation between the stage of reductive evolution and repeat content (17). This is likely because deletions and pseudogenization impact fitness as more genes become nonfunctional, and the remaining genes become indispensable. Therefore, it has been suggested that repeats might be involved in the early phase of reductive evolution and that a different mechanism is responsible for the gradual deletion of pseudogenes (17). *M. lepraemurium* has only a few repeats (Table S1), which mostly derive from three families of dysfunctional insertion sequences (IS), each consisting of up to six copies.

10.1128/mBio.01283-17.5 TABLE S1  *M. lepraemurium* repetitive genomic regions. Download TABLE S1, DOCX file, 0.01 MB. Copyright © 2017 Benjak et al. 2017 Benjak et al. This content is distributed under the terms of the Creative Commons Attribution 4.0 International license.

Curiously, one IS family consisting of six copies (*MLM_1414*, *MLM_1873*, *MLM_2691*, *MLM_2913*, *MLM_3078*, and *MLM_3876*) shows 84% nucleotide identity with the ISMsm2 mobile element from *Mycobacterium smegmatis* (NCBI accession number WP_003887303). A BLAST search showed that this IS family resides in only a few other unrelated mycobacteria and not in the MAC, with the exception of a more diverged copy (with 78% identity) present in the plasmid of *M. avium* subsp. *hominissuis* strain 88Br (GenBank accession number KR997898.1) and a truncated copy (with 81% identity) in the genome of *M. avium* subsp. *hominissuis* strain H87 (GenBank accession number CP018363.1). Strikingly, when we analyzed the genomic synteny between *M. lepraemurium*, *M. intracellulare*, and *M. avium* subsp. *hominissuis*, all the synteny breaks in *M. lepraemurium* occurred at the locations of this particular IS (Fig. S3A and B). The synteny plot between *M. lepraemurium* and *M. avium* subsp. *paratuberculosis* looked different from the aforementioned genomes, which is probably due to genomic rearrangements in *M. avium* subsp. *paratuberculosis* (Fig. S3C).

10.1128/mBio.01283-17.4 FIG S3  Synteny plots between *M. lepraemurium* and selected members of the *Mycobacterium avium* complex. Genome sequences are represented as colored ideograms. Numbered ticks mark million bases. Transposable elements are shown as ticks in the second track, with the ISMsm2-like family in magenta. Inner links are LAST hits (see Text S1 in the supplemental material for details). Download FIG S3, TIF file, 2.8 MB. Copyright © 2017 Benjak et al. 2017 Benjak et al. This content is distributed under the terms of the Creative Commons Attribution 4.0 International license.

The presence of identical copies of dysfunctional transposable elements indicates that *M. lepraemurium* lost functional transposases relatively recently. As mentioned above, at least one IS family was responsible for large genomic rearrangements in *M. lepraemurium*, and a deeper analysis of the genome sequence might shed light on the driving force that caused major deletions in this genome.

### *M. lepraemurium*-specific sequences.

Comparison of *M. lepraemurium* with other members of the MAC revealed a total of 32.2 kb of genomic sequence that was not found in any publicly available genome sequence. These regions contain 35 genes, of which only 12 are functional (Table S2). One of these genes, *MLM_3300*, codes for a Fic family protein. Fic proteins have been associated with pathogenicity in bacteria, often acting as toxins that interfere with the host cell in different ways (18).

10.1128/mBio.01283-17.6 TABLE S2  *M. lepraemurium*-specific genomic regions and genes. Download TABLE S2, DOCX file, 0.01 MB. Copyright © 2017 Benjak et al. 2017 Benjak et al. This content is distributed under the terms of the Creative Commons Attribution 4.0 International license.

### Interaction with macrophages.

After entering the macrophage, members of the MAC reside within phagosomes (19). There they inhibit phagosome maturation by preventing acidification to a pH below 6.4, and thus, prevent fusion with the extremely acidic lysosome. In *M. avium*, mutations in the PPE gene *MAV_2928* and the PE gene *MAV_1346* inhibit maturation and acidification of phagosomes, resulting in decreased virulence (20, 21). In *M. lepraemurium*, gene *MLM_2357* (homologous to *MAV_2928*) and *MLM_1265* (homologous to *MAV_1346*) are functional, suggesting that they may help its survival in macrophages. Additionally, the gene *MLM_2012* encodes the lipoprotein, LppM, which is an important virulence factor in *M. tuberculosis* and interferes with phagosomal maturation in macrophages (22).

Reactive oxygen species (ROS) are produced by the macrophage as a bactericidal mechanism in response to infection. The ability of pathogens to produce enzymes such as catalase-peroxidase, epoxide hydrolase, and superoxide dismutase (SOD), which remove ROS, enable their survival within macrophages. While it has been suggested that *M. lepraemurium* abolishes the production of ROS upon entering the macrophage (29), probably as a survival mechanism, it remains unclear whether small quantities of ROS are produced upon infection and handled by the bacterium. *M. lepraemurium* has retained four functional epoxide hydrolases (*MLM_0642*, *MLM_0684*, *MLM_1194*, and *MLM_1485*) and one catalase-peroxidase (*MLM_2092*), which explains its observed catalase-peroxidase activity (23). Moreover, *M. lepraemurium* is able to produce two superoxide dismutases, SodA and SodC (*MLM_0123* and *MLM_2650*).

### Glycopeptidolipid synthesis.

Glycopeptidolipids (GPLs) are synthesized by several nontuberculosis mycobacteria, including members of the MAC. GPLs are present on the outermost layer of the cell wall, and therefore, they play an important role in sliding motility, biofilm formation, and pathogenesis (24). This is even more relevant within the MAC, whose members can produce a number of serovar-specific variations of GPLs. The *M. lepraemurium* cell wall contains glycolipids and amino acids (25, 26); however, production of GPLs in *M. lepraemurium* has not been studied. Most of the genes known to be involved in the biosynthesis of non-serovar-specific GPLs are intact in *M. lepraemurium*, albeit with a few exceptions (Table S3). Although it is difficult at this moment to predict the effects of these mutations, it appears that *M. lepraemurium* should be able to produce GPLs, as all of the core genes are present.

10.1128/mBio.01283-17.7 TABLE S3  Genes implicated in the biosynthesis of GPLs in the *Mycobacterium avium* complex. Download TABLE S3, DOCX file, 0.02 MB. Copyright © 2017 Benjak et al. 2017 Benjak et al. This content is distributed under the terms of the Creative Commons Attribution 4.0 International license.

### Virulence.

While the ESX-1 system is the main determinant of virulence in *M. tuberculosis* and *M. leprae* (27), it is absent in the MAC. However, ESX-5, which is also associated with virulence in pathogenic mycobacteria, is mostly intact in *M. lepraemurium*, except for the cytochrome P450 hydroxylase (*MLM_2361*), which is also nonfunctional in *M. leprae*. The duplicated four-gene region, ESX-5a, encoding EsxI, EsxJ, a PPE, and a PE protein may serve as an accessory system for transport of a subset of ESX-5 proteins (28) and is still functional in *M. lepraemurium*. In *M. lepraemurium*, the PPE and PE genes are merged into a single open reading frame, as in *M. avium* subsp. *paratuberculosis* but not in other members of the MAC. An interesting observation is that in MAC, *esxI* and *esxJ* are 100% identical to *esxN* and *esxM* from the main ESX-5 locus (unlike in *M. tuberculosis*), which indicates a novel and recent duplication event and suggests that a crucial function might lie behind redundancy of the ESX-5 components.

The *pks12* gene is involved in the biosynthesis of mannosyl-β-1-phosphomycoketides (MPM) and is found only in the slow-growing mycobacteria. In different pathogenic mycobacteria, including *M. avium* (20), *pks12* was shown to be necessary for the virulence, and this gene is functional in *M. lepraemurium* (*MLM_2156*).

### Experimental procedures.

*M. lepraemurium* strain Hawaii was grown in BALB/c mice. The DNA was sequenced using Illumina and PacBio technologies, followed by sequence assembly and annotation. More details are given in Text S1 in the supplemental material.

10.1128/mBio.01283-17.1 TEXT S1  Supplemental Materials and Methods. Download TEXT S1, DOCX file, 0.03 MB. Copyright © 2017 Benjak et al. 2017 Benjak et al. This content is distributed under the terms of the Creative Commons Attribution 4.0 International license.

### Accession number(s).

The annotated genome was submitted to GenBank under GenBank accession number CP021238.
