# Supplementary material for: Insights from the Genome Sequence of Mycobacterium lepraemurium: Massive Gene Decay and Reductive Evolution
Source: mBio. 2017 Oct 17;8(5):e01283-17. doi: 10.1128/mBio.01283-17 (PMC5646247; doi:10.1128/mBio.01283-17)
Supplement: TABLE S1 [file mbo005173527st1.docx]

**Table S1: *M. lepraemurium* repetitive genomic regions.**

| Repeat cluster | Start | End | Length | Gene(s) | Pseudogene | Annotation |
| --- | --- | --- | --- | --- | --- | --- |
| 1 | 2590876 | 2593150 | 2275 | MLM_2604 | no | non-ribosomal peptide synthetase 1 |
|  | 2595255 | 2597610 | 2356 | MLM_2605 | no | non-ribosomal peptide synthetase 2 |
|  | 2599761 | 2602116 | 2356 | MLM_2605 | no | non-ribosomal peptide synthetase 2 |
| 2 | 2104068 | 2105674 | 1607 | MLM_2156 | no | malonyl CoA-acyl carrier protein transacylase |
|  | 2110167 | 2111773 | 1607 | MLM_2156 | no | malonyl CoA-acyl carrier protein transacylase |
| 3 | 1337854 | 1339349 | 1496 | MLM_1358 | yes | partial REP13E12 repeat protein |
|  | 3383223 | 3384718 | 1496 | MLM_3341 | yes | uncharacterized protein (HNH endonuclease, 13E12 repeat family) |
|  | 3520566 | 3522018 | 1453 | MLM_3478 | yes | REP13E12 repeat protein (fragment) |
| 4 | 672122 | 673674 | 1553 | MLM_0680 | yes | mobile element protein |
|  | 1596190 | 1597583 | 1394 | MLM_1641 | yes | IS481 family transposase |
|  | 1998427 | 1999979 | 1553 | MLM_2038 | yes | mobile element protein |
| 5 | 2000175 | 2001365 | 1191 | MLM_2040 | yes | partial REP13E12 repeat protein |
|  | 2200411 | 2201601 | 1191 | MLM_2245 | yes | partial REP13E12 repeat protein |
| 6 | 1398771 | 1399903 | 1133 | MLM_1414 | yes | mobile element protein |
|  | 1832525 | 1833069 | 545 | MLM_1873 | yes | mobile element protein |
|  | 2707760 | 2708895 | 1136 | MLM_2691 | yes | mobile element protein |
|  | 2962176 | 2963335 | 1160 | MLM_2913 | yes | mobile element protein |
|  | 3125465 | 3125919 | 455 | MLM_3078 | yes | mobile element protein |
|  | 3896248 | 3897425 | 1178 | MLM_3876 | yes | mobile element protein |
| 7 | 1876090 | 1877213 | 1124 | MLM_1920 | no | 3-oxoacyl-[acyl-carrier-protein] synthase |
|  | 3545427 | 3546550 | 1124 | MLM_3503 | no | 3-oxoacyl-[acyl-carrier-protein] synthase |
| 8 | 129992 | 131015 | 1024 | MLM_0130 | yes | mobile element protein |
|  | 959494 | 960036 | 543 | MLM_0985 | yes | mobile element protein |
|  | 1927549 | 1928568 | 1020 | MLM_1967A | yes | mobile element protein |
|  | 2561454 | 2562473 | 1020 | MLM_2584 | yes | mobile element protein |
|  | 3170319 | 3171327 | 1009 | MLM_3126 | yes | mobile element protein |
|  | 3609141 | 3610160 | 1020 | MLM_3568 | yes | mobile element protein |
| 9 | 1101447 | 1102085 | 639 | MLM_1123 + MLM_1124 | no | EsxI + EsxJ |
|  | 2311416 | 2312054 | 639 | MLM_2353 + MLM_2354 | no | EsxN + EsxM |
| 10 | 3382732 | 3383042 | 311 | MLM_3339 | no | hypothetical protein |
|  | 3383043 | 3383353 | 311 | MLM_3341 | yes | uncharacterized protein (HNH endonuclease, 13E12 repeat family) |
