# Supplementary figures and images for: Insights from the Genome Sequence of Mycobacterium lepraemurium: Massive Gene Decay and Reductive Evolution
Source: mBio. 2017 Oct 17;8(5):e01283-17. doi: 10.1128/mBio.01283-17 (PMC5646247; doi:10.1128/mBio.01283-17)

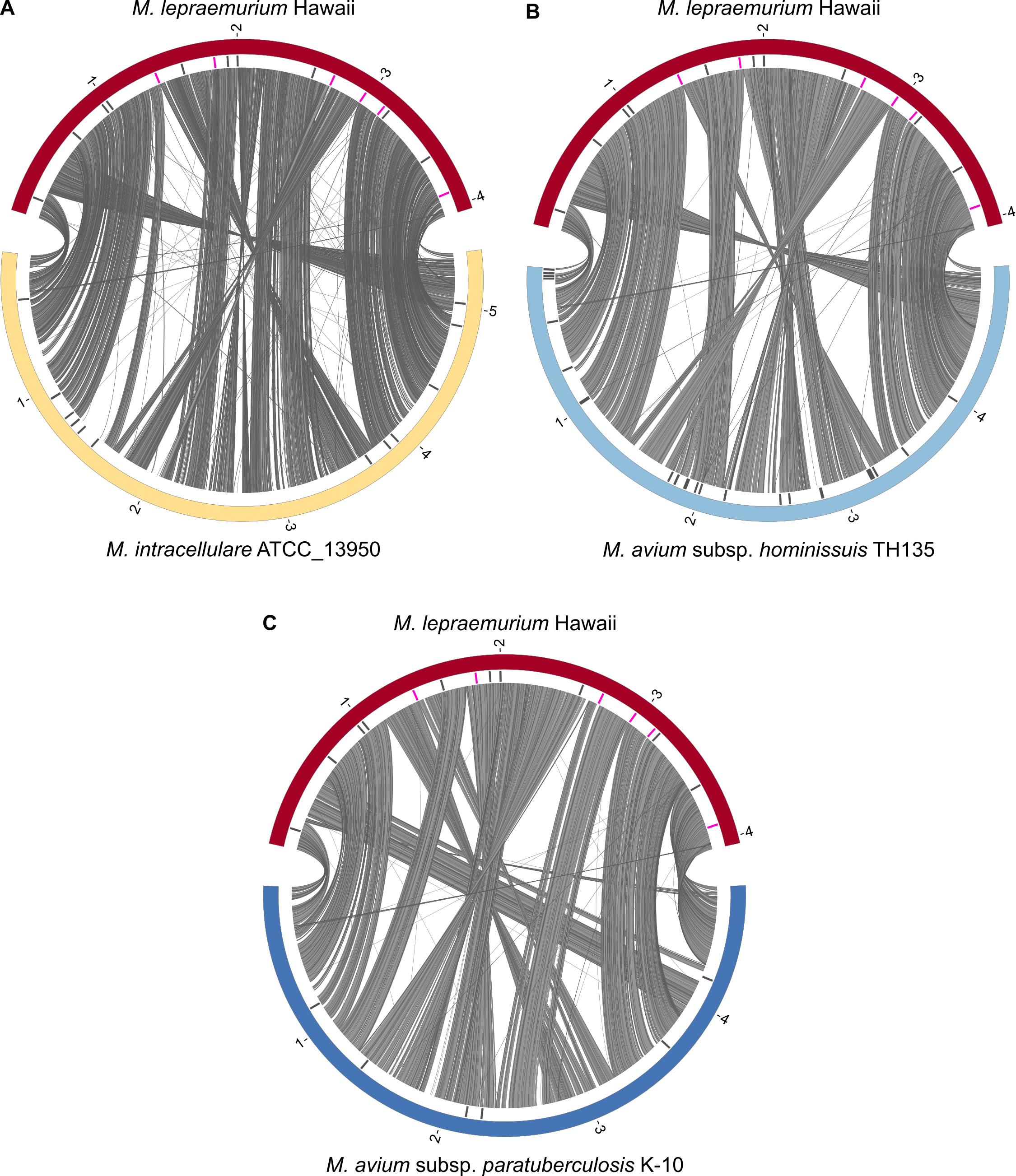

Supplement: FIG S3 [file mbo005173527sf3.tif]
