# Supplementary material for: Insights from the Genome Sequence of Mycobacterium lepraemurium: Massive Gene Decay and Reductive Evolution
Source: mBio. 2017 Oct 17;8(5):e01283-17. doi: 10.1128/mBio.01283-17 (PMC5646247; doi:10.1128/mBio.01283-17)
Supplement: TABLE S2 [file mbo005173527st2.docx]

**Table S2: *M. lepraemurium* specific genomic regions and genes.**

| Genomic region | Length | Overlapping gene | Start | Stop | Product | Function |
| --- | --- | --- | --- | --- | --- | --- |
| 954701‑ 956100 | 1400 | MLM_0980 | 954318 | 955445 | FAD-binding monooxygenase | Protein‑coding |
|  |  | MLM_0981 | 955549 | 956094 | TetR family transcriptional regulator | Protein‑coding |
| 1039201‑ 1039800 | 600 | NA | NA | NA | NA | NA |
| 1042001‑ 1042600 | 600 | MLM_1065 | 1042056 | 1042322 | Helix-turn-helix XRE-family transcriptional regulator | Pseudogene |
|  |  | MLM_1066 | 1042397 | 1042630 | hypothetical protein | Protein‑coding |
| 1308101‑ 1308400 | 300 | MLM_1327 | 1308059 | 1308490 | putative extradiol dioxygenase | Protein‑coding |
| 1443701‑ 1444000 | 300 | MLM_1462 | 1443654 | 1444794 | uncharacterized protein | Pseudogene |
| 1789901‑ 1792100 | 2200 | MLM_1829 | 1789973 | 1790841 | restriction endonuclease | Pseudogene |
|  |  | MLM_1829A | 1791008 | 1791931 | hypothetical protein | Protein‑coding |
| 2022601‑ 2024200 | 1600 | MLM_2063 | 2022685 | 2023435 | short-chain dehydrogenase | Pseudogene |
|  |  | MLM_2065 | 2023485 | 2024215 | putative short-chain dehydrogenase | Pseudogene |
| 2707801‑ 2721800 | 14000 | MLM_2691 | 2707807 | 2708890 | mobile element protein | Pseudogene |
|  |  | MLM_2693 | 2708891 | 2709731 | aldo-keto reductase | Pseudogene |
|  |  | MLM_2695 | 2709960 | 2710996 | putative oxidoreductase YncB | Pseudogene |
|  |  | MLM_2696 | 2711067 | 2711455 | putative LysR-family transcriptional regulator | Pseudogene |
|  |  | MLM_2697 | 2711736 | 2712220 | putative LysR-family transcriptional regulator | Pseudogene |
|  |  | MLM_2698 | 2712184 | 2712453 | TetR family transcriptional regulator | Pseudogene |
|  |  | MLM_2699 | 2712541 | 2713507 | quinone oxidoreductase | Pseudogene |
|  |  | MLM_2701 | 2713577 | 2714488 | putative LysR-family transcriptional regulator | Protein‑coding |
|  |  | MLM_2702 | 2714586 | 2715407 | hydroxymethylglutaryl-CoA lyase | Protein‑coding |
|  |  | MLM_2703 | 2715424 | 2715951 | acyl dehydratase | Protein‑coding |
|  |  | MLM_2704 | 2715951 | 2717291 | 4-hydroxybutyrate:acetyl-CoA CoA transferase | Protein‑coding |
|  |  | MLM_2705 | 2717306 | 2719057 | pyruvate oxidase | Protein‑coding |
|  |  | MLM_2706 | 2719437 | 2719946 | nitroreductase | Protein‑coding |
|  |  | MLM_2707 | 2720165 | 2720832 | short-chain dehydrogenase/reductase | Pseudogene |
| 3121401‑ 3129500 | 8100 | MLM_3070 | 3121408 | 3121638 | uncharacterized protein | Pseudogene |
|  |  | MLM_3071 | 3122087 | 3123147 | uncharacterized protein | Pseudogene |
|  |  | MLM_3074 | 3123144 | 3124244 | uncharacterized protein | Pseudogene |
|  |  | MLM_3077 | 3124699 | 3125469 | DUF58 domain-containing protein | Pseudogene |
|  |  | MLM_3078 | 3125469 | 3125887 | mobile element protein | Pseudogene |
|  |  | MLM_3079 | 3126127 | 3127092 | moxR-like ATPases | Pseudogene |
|  |  | MLM_3080 | 3127190 | 3129141 | uncharacterized protein | Pseudogene |
| 3341401‑ 3342500 | 1100 | MLM_3300 | 3341340 | 3342548 | Fic family protein | Protein‑coding |
| 3552401‑ 3552700 | 300 | MLM_3510 | 3552383 | 3552748 | uncharacterized protein | Pseudogene |
| 3569501‑ 3571200 | 1700 | MLM_3526 | 3569444 | 3570305 | uncharacterized protein | Pseudogene |
|  |  | MLM_3527 | 3570428 | 3571137 | uncharacterized protein | Pseudogene |
