# Supplementary material for: Insights from the Genome Sequence of Mycobacterium lepraemurium: Massive Gene Decay and Reductive Evolution
Source: mBio. 2017 Oct 17;8(5):e01283-17. doi: 10.1128/mBio.01283-17 (PMC5646247; doi:10.1128/mBio.01283-17)
Supplement: TABLE S3 [file mbo005173527st3.docx]

**Table S3: Genes implicated in the biosynthesis of GPLs in the *Mycobacterium avium* complex.**

| **Gene name(s)** | **Product and/or function** | ***M. avium* 104** | ***M. colombiense* CECT_3035** | ***M. intracellulare* ATCC_13950** | ***M. lepraemurium* Hawaii** |
| --- | --- | --- | --- | --- | --- |
| *mmps4* | Required for assembly of GPL synthesis enzymes in the cell membrane | MAV_3247 | MCOL_V218616 | OCU_30810 | MLM_2607 |
| *mmpL4a* | Required for assembly of GPL biosynthases in the cell membrane | MAV_3248 | MCOL_V218621 | OCU_30820 | MLM_2608 |
| *mmpL4b* | Required for assembly of GPL biosynthases in the cell membrane | MAV_3249 | MCOL_V218626 | OCU_30830 | MLM_2609 |
| *MAV_3362* | Unknown function | MAV_3362 | MCOL_V219166 | OCU_32010 | MLM_1724 |
| *rmlA* | Glucose-1-phosphate thymidylyltransferase | MAV_4820 | MCOL_V201710 | OCU_46990 | MLM_3982 |
| *rmlB* | NAD dependent epimerase/dehydratase family protein | MAV_3269 | MCOL_V218756 | OCU_31110 | MLM_2632 |
| *mtfA* | 3-O-methyltransferase | MAV_3268 | WP_075235767.1* | OCU_27880 | MLM_2631 (pseudogene) |
| *mtfB* | Rhamnose 4-O-methyltransferase | MAV_3266 | MCOL_V218751 | OCU_31100 | MLM_2630 |
| *mtfC* | Rhamnose 4-O-methyltransferase | MAV_3261 | MCOL_V218736 | OCU_31070 | MLM_2626 |
| *mtfD* | Rhamnose 3-O-methyltransferase | MAV_3260 | MCOL_V218731 | OCU_31060 | MLM_2625 |
| *gtfA* | Glycosyl transferase family protein | MAV_3265 | MCOL_V218746 | OCU_31090 | MLM_2629 |
| *gtfB* | Glycosyl transferase family protein | MAV_3258 | WP_065074816.1** | OCU_31040 | MLM_2623 |
| *gtfD* | Putative glycosyl transferase | MAV_3253 | WP_075237225.1* | OCU_30870 | Not found |
| *rtfA* | Putative glycosyl transferase | MAV_3262 | MCOL_V218741 | OCU_31080 | MLM_2627 |
| *atf* | Putative acyltransferase | MAV_3274 | MCOL_V218771 | OCU_31140 | MLM_2635 (pseudogene) |
| *gplH* | Unknown function | MAV_3245 | MCOL_V218611 | OCU_30790 | MLM_2606 |
| *pstA (mps1)* | Linear gramicidin synthetase subunit D | MAV_3244 | MCOL_V218606 | OCU_30780 | MLM_2605 |
| *pstB (mps2)* | Linear gramicidin synthetase subunit B | MAV_3243 | MCOL_V218601 | OCU_30770 | MLM_2604 |
| *gap* | Required for GPL export. | MAV_3059 | MCOL_V222543 | OCU_28790 | MLM_2430 (pseudogene) + MLM_2431 (pseudogene) |
| *sap* | Dgpf domain family protein | MAV_4518 | MCOL_V200150 | OCU_43890 | MLM_3677 |
| *ecf* | RNA polymerase ECF-subfamily protein sigma factor | MAV_4519 | MCOL_V200155 | OCU_43900 | MLM_3678 |
| *fadE5* | Putative acyl-CoA dehydrogenase | MAV_3309 | MCOL_V218931 | OCU_31500 | MLM_1767 (pseudogene) |
| *MAV_2461* | Uncharacterized protein | MAV_2461 | MCOL_V208010 | OCU_24450 | MLM_2147 (Partial hit. Also, the ORF is much longer) |
| *pks* | Polyketide synthases | MAV_1763 | MCOL_V205565 | OCU_18380 | MLM_2746 |
| *papA3* | Condensation domain protein | MAV_1762 | MCOL_V205560 | OCU_18360 | MLM_2749 |
| *mmpL10* | Transport protein | MAV_1761 | MCOL_V205550 | OCU_18350 | MLM_2750 |
| *fadD23* | acyl-CoA synthase | MAV_1759 | WP_081293032.1* | OCU_18330 | MLM_2752 |
| *pe* | Uncharacterized protein | MAV_1760 | WP_065054842.1* | OCU_18340 | MLM_2751 |
| *MAV_1758* | Uncharacterized protein | MAV_1758 | WP_076099905.1* | OCU_12310 | MLM_2753 |
| *dhgA* | Dehydrogenase DhgA | MAV_3259 | Not found | OCU_31050 | MLM_2624 |

*As in *M. avium* 104

**The gene is absent in *M. colombiense* CECT_3035, but is present in other *M. colombiense* strains. A representative protein is given.

***The gene is present in *M. colombiense* CECT_3035, but is not annotated. A representative protein from another strain is given.
