# Supplementary material for: Insights from the Genome Sequence of Mycobacterium lepraemurium: Massive Gene Decay and Reductive Evolution
Source: mBio. 2017 Oct 17;8(5):e01283-17. doi: 10.1128/mBio.01283-17 (PMC5646247; doi:10.1128/mBio.01283-17)
Supplement: TEXT S1 [file mbo005173527s1.docx]

**Insights from the genome sequence of *Mycobacterium lepraemurium*: massive gene decay and reductive evolution**

**Andrej Benjak, Tanvi P. Honap, Charlotte Avanzi, Luis E. Becerril-Villanueva, Iris Estrada-García, Oscar Rojas-Espinosa, Anne C. Stone and Stewart T. Cole**

**SUPPLEMENTARY MATERIALS AND METHODS**

**Bacilli culture and purification.** *M. lepraemurium* Hawaii was grown using serial infections in BALB/c mice injected by the intraperitoneal route. At four to six months post-infection, the infected spleen and liver were harvested. Bacteria were purified by following the protocol in (1), followed by the Percoll step (2) and then by using previously established protocols (3, 4). Briefly, 4 g of tissue was suspended in 20 ml of 0.2 M sucrose and ground in a glass Potter–Elvehjem homogenizer. The resulting suspension was centrifuged for 20 min at 150 xg to separate cell debris (Sorvall RC5B, rotor HB4, Sorvall Instruments, Wilmington, Delaware, USA). Then, 9 ml of the isolated supernatant was overlaid onto 12 ml of 0.3 M sucrose and the tubes were centrifuged at 3,500 xg for 10 min at 4–10°C (Sorvall RC5B, rotor SS34). The resulting bacilli-rich pellet was resuspended in 20 ml of 0.2 M sucrose and overlaid, in 9-ml aliquots, onto 12 ml of 1.5 M KCl. The tube was then centrifuged at 4°C for 10 min at 3,500 xg. The bacilli were collected, washed 3 times with phosphate-buffered-saline (PBS) at pH 7.4 (PBS is 0.01 M Na/K phosphate, 0.15 M NaCl), and suspended in 40 ml of a solution containing a mixture of Percoll (3 parts) and 0.1% Tween 80 (7 parts). The suspension was centrifuged at 23,000 xg (Sorvall RC5B, rotor SS34) for 60 min at 40°C. Then the bacillary layer was resuspended in 20 ml of Percoll-Tween and centrifuged as before. The final bacillary pellet was collected and washed 5 times with PBS pH 7.4 or until the Percoll was completely eliminated. The purity of the bacillary preparation was verified by Ziehl–Neelsen staining. The purified bacillary suspension was prepared in synthetic 7H9 Middlebrook broth-OADC medium (DIFCO, Detroit, MI, USA), and quantified via a nephelometric reference curve prepared with known quantities of bacteria. The bacillary suspension was aliquoted and frozen at -20°C until ready for use.

For DNA extraction, several aliquots were combined, centrifuged at 3,500 xg for 10 min, the supernatant was eliminated and the pellet was frozen (-20°C) without any further treatment.

**DNA extraction.** DNA extraction was carried out using a custom-designed protocol for mycobacterial DNA. The bacterial cell pellet was washed with 500 µL of phosphate buffer saline (PBS) prior to centrifugation at 5000 g for 10 min. The supernatant was discarded and the pellet was re-suspended in 1 mL of bacterial lysis buffer B1 (50 mM Tris-HCl pH 8.0; 50 mM EDTA pH 8.0; 0.5% Tween 20; 0.5% Triton-X100) containing 45 µL of proteinase K (20 mg/mL) and 20 µL of lysozyme (100 mg/mL). The mixture was then transferred into bead-beating tubes containing 500 μL of silica beads (0.1 mm zirconia beads) prior to physical disruption using the Precellys24 homogenizer at 6.5 m/s for 25 sec. After incubating at 56°C for 1 h, the mixture was centrifuged and the supernatant was transferred to a new tube. An additional incubation with 20 µL proteinase K (20 mg/mL) was conducted at 56°C for 30 min. The mixture was then incubated at 4°C for 15 min. RNAse A (Sigma) was added and the sample was incubated 30 min at 37°C, followed by the addition of 350 µL of bacterial lysis buffer B2 (3M guanidine hydrochloride, 20% Tween 20), and incubated for 30 min at 50°C. DNA was purified using the Qiagen Genomic-Tip/20G according to the manufacturer’s instructions, and eluted in 2 mL elution buffer. The DNA was precipitated using 0.7x volume of isopropanol and centrifuged at 4°C for 15 min. The pellet was washed twice with 200 µL 70% ethanol, air-dried, and suspended overnight in 200 µL Tris HCl buffer (pH 8.0) at room temperature under continuous shaking. The DNA was then purified using AMPure beads (Thermofisher) at a ratio of 0.45. The quality of the DNA extract was checked using the Fragment Analyzer (Advanced Analytical Technologies) and quantified using the Qubit 2.0 (Life Technologies).

**Illumina sequencing.** DNA (50 μL) was sheared using the Covaris S220 Focused-ultrasonicator (Covaris) to obtain 400 bp-long DNA fragments, and purified using AMPure beads (1.8x) and the manufacturer’s protocol. The sheared DNA was quantified using the dsDNA High Sensitivity assay and the Qubit 2.0 flurometer (Life Technologies). Up to 1 μg of DNA in 50 μL was used for library preparation using the Kapa Hyper prep kit (Roche) and PentAdapters (Pentabase) for indexing. The library was quantified using the dsDNA Broad Range assay and the Qubit 2.0 fluorometer. The library was sequenced on an Illumina HiSeq 2500 (1 x 101 bp run).

**PacBio sequencing.** DNA (5.1 µg) was sheared using a Covaris g-TUBE (Covaris S220) to obtain 10 kb fragments and the size distribution was checked using the Fragment Analyzer (Advanced Analytical Technologies). Sheared DNA (4 µg) was used to prepare a SMRTbell library with the PacBio SMRTbell Template Prep Kit 1 (Pacific Biosciences) according to the manufacturer's recommendations. The resulting library was size-selected using a BluePippin system (Sage Science, Inc.) for molecules larger than 8 kb. The recovered library was sequenced using a SMRT cell with P6/C4 chemistry and MagBeads on a PacBio RSII system (Pacific Biosciences) at 240 min movie length.

**Genome assembly.** PacBio reads were processed using the HGAP2 and HGAP3 pipelines (5). The resulting contigs were compared to the nucleotide database at NCBI using BLAST (6). The two largest contigs produced by HGAP3 v2.3.0 (which were 2.3 and 1.7 Mb in length, respectively) matched to *M. avium* sequences. These two contigs corresponded to the three largest contigs produced by HGAP2 v2.3.0 (which were 1.7, 1.6, and 0.6 Mb in length) and two shorter contigs (61 and 21 kb in length). The two HGAP3 contigs could be joined by the overlapping HGAP2 contigs, resulting in a single consensus sequence with overlapping ends, indicative of a circular genome. To correct for possible sequence errors, Illumina reads were mapped onto the draft genome sequence using Bowtie2 (7) resulting in 35-fold coverage of non-duplicate reads. Variants were called using SAMtools mpileup (Li et al., 2009) and VarScan2 (9), resulting in only five single nucleotide polymorphisms (SNPs) and two short insertion-deletions (InDels).

Illumina reads (4%) that did not map to the final genome sequence were assembled using MIRA (https://sourceforge.net/projects/mira-assembler/). The resulting 34 contigs (of which the largest was 1.9 kbp long) were compared to the nucleotide and protein databases at NCBI using BLAST. The contigs matched to *Mus musculus* or to various bacteria. No evidence of a putative plasmid sequence was found.

**Gene prediction.** *De novo* gene prediction was conducted using the RAST server (10) with the frameshift correction option. Reference-based gene prediction was conducted using RATT (11) with annotations from *M. avium* subsp. *paratuberculosis* K-10 (NC_002944.2) and *M. avium* subsp. *hominissuis* TH135 (AP012555.1). All predictions were merged, and inconsistencies and large intergenic areas were manually checked by using BLAST to compare the problematic sequences against the protein database at NCBI. Gene predictions, shorter than 100 nucleotides in length and not conserved in the genomes of other *M. avium* species, were removed. The annotated genome was submitted to GenBank (accession number CP021238).

**Phylogenetic analyses.** Two different strategies were used for the phylogenetic analysis to assure accuracy and consistency of the reconstructed tree topologies, as described below.

**Phylogenetic analysis of concatenated amino-acid sequences.** A set of eleven genes was chosen for the analysis (12), (DnaN, RplI, GrpE, MetG, RplY, PheT, FtsQ, HolA, MiaA, FtsY, FtsX). To make sure to identify strains that are closely related to *M. lepraemurium*, the nucleotide sequence of *M. lepraemurium* corresponding to the abovementioned genes was used as dcBLASTn query against all available genome sequences and genome assemblies of the MAC available at NCBI as of June 2017. BLAST hits were translated into amino-acid sequence. Amino-acid sequences of additional genomes from the MAC complex as well as other mycobacteria were included. Analysis of the concatenated alignments was done in MEGA7 (13).

**Phylogenetic analysis of whole-genome alignments.** Publically available genome data were acquired for comparative purposes for 16 mycobacterial species. Contigs or finished genomes of these species were aligned to the *M. avium* 104 reference genome using LAST (14) with the following parameters: -u = 0, -e = 34, and -j = 5. The maf-convert program was used to covert the alignment file to a SAM file and SAMtools was used obtain a BAM file which was used for further analyses. SAMtools mpileup and bcftools call were used to produce the VCF files. VCF files for all strains were combined using the CombineVariants tool available in the Genome Analysis Toolkit (GATK) (15). The SelectVariants tool in GATK was used to output a VCF file containing the sites comprising SNPs. VCFtools (16) was used to remove InDels, tri-allelic sites, and sites with missing data. An SNP alignment was generated using a publically available perl script (17), which comprised a total of 460,625 sites.

Phylogenetic trees were constructed using the Maximum Likelihood (ML) method in RAxML v7.2.8 (18) and the Neighbor-Joining (NJ) and Maximum Parsimony (MP) methods in MEGA7 (13). The ML tree was generated using the GTR-GAMMA model and 100 bootstrap replicates (Figure 1). The NJ tree was generated using the p-distance method and bootstrap support was estimated from 500 replicates (Figure S1). The MP tree was generated using the Subtree-Pruning-Regrafting (SPR) algorithm and 500 bootstrap replicates (Figure S2).

**Comparison of orthologous genes.** Orthologs between *M. lepraemurium* and *M. avium* subsp. *paratuberculosis* were inferred by RATT and manually during the annotation process. Orthologs between *M. avium*, *M. leprae* and *M. ulcerans* were retrieved from <http://www.pathogenomics.sfu.ca/ortholugedb/>.

**References:**

1. Prabhakaran K, Harris EB, Kirchheimer WF. 1976. Binding of 14C labeled dopa by Mycobacterium leprae in vitro. Int J Lepr 44:58–64.

2. Draper P. 1980. Purification of Mycobacterium lepraeReport of the Fifth Meeting of the Scientific Working Group on the Immunology of Leprosy, TDR/IMMLEP‑SWG 5/80.3.

3. Wek-Rodriguez K, Silva-Miranda M, Arce-Paredes P, Rojas-Espinosa O. 2007. Effect of reactive oxygen intermediaries on the viability and infectivity of Mycobacterium lepraemurium. Int J Exp Pathol 88:137–145.

4. Rojas-Espinosa O, Wek-Rodriguez K, Arce-Paredes P. 2002. The effect of exogenous peroxidase on the evolution of murine leprosy. IntJLeprOther MycobactDis 70:191–200.

5. Chin C-S, Alexander DH, Marks P, Klammer AA, Drake J, Heiner C, Clum A, Copeland A, Huddleston J, Eichler EE, Turner SW, Korlach J. 2013. Nonhybrid, finished microbial genome assemblies from long-read SMRT sequencing data. Nat Methods 10:563–569.

6. Altschul SF, Gish W, Miller W, Myers EW, Lipman DJ. 1990. Basic local alignment search tool. J Mol Biol 215:403–410.

7. Langmead B, Salzberg SL. 2012. Fast gapped-read alignment with Bowtie 2. Nat Methods 9:357–359.

8. Li H, Handsaker B, Wysoker A, Fennell T, Ruan J, Homer N, Marth G, Abecasis G, Durbin R. 2009. The Sequence Alignment/Map format and SAMtools. Bioinformatics 25:2078–2079.

9. Koboldt DC, Zhang Q, Larson DE, Shen D, McLellan MD, Lin L, Miller CA, Mardis ER, Ding L, Wilson RK. 2012. VarScan 2: Somatic mutation and copy number alteration discovery in cancer by exome sequencing. Genome Res 22:568–576.

10. Aziz RK, Bartels D, Best AA, DeJongh M, Disz T, Edwards RA, Formsma K, Gerdes S, Glass EM, Kubal M, Meyer F, Olsen GJ, Olson R, Osterman AL, Overbeek RA, McNeil LK, Paarmann D, Paczian T, Parrello B, Pusch GD, Reich C, Stevens R, Vassieva O, Vonstein V, Wilke A, Zagnitko O. 2008. The RAST Server: Rapid Annotations using Subsystems Technology. BMC Genomics 9:75.

11. Otto TD, Dillon GP, Degrave WS, Berriman M. 2011. RATT: Rapid Annotation Transfer Tool. Nucleic Acids Res 39:e57.

12. Mizuno T, Natori T, Kanazawa I, Eldesouky I, Fukunaga H, Ezaki T. 2016. Core housekeeping proteins useful for identification and classification of mycobacteria 32.

13. Kumar S, Stecher G, Tamura K. 2016. MEGA7: Molecular Evolutionary Genetics Analysis Version 7.0 for Bigger Datasets. Mol Biol Evol 33:1870–1874.

14. Kiełbasa SM, Wan R, Sato K, Horton P, Frith MC. 2011. Adaptive seeds tame genomic sequence comparison. Genome Res 21:487–493.

15. McKenna A, Hanna M, Banks E, Sivachenko A, Cibulskis K, Kernytsky A, Garimella K, Altshuler D, Gabriel S, Daly M, DePristo MA. 2010. The Genome Analysis Toolkit: a MapReduce framework for analyzing next-generation DNA sequencing data. Genome Res 20:1297–303.

16. Danecek P, Auton A, Abecasis G, Albers CA, Banks E, DePristo MA, Handsaker RE, Lunter G, Marth GT, Sherry ST, McVean G, Durbin R. 2011. The variant call format and VCFtools. Bioinformatics 27:2156–2158.

17. Bergey C. 2012. vcf-tab-to-fasta.

18. Stamatakis A. 2006. RAxML-VI-HPC: Maximum Likelihood-based Phylogenetic Analyses with Thousands of Taxa and Mixed Models. Bioinformatics 22:2688–2690.
